# Transcriptomic Complexity in Strawberry Fruit Development and Maturation Revealed by Nanopore Sequencing

**DOI:** 10.3389/fpls.2022.872054

**Published:** 2022-07-13

**Authors:** Qing Chen, Ximeng Lin, Wenlu Tang, Qian Deng, Yan Wang, Yuanxiu Lin, Wen He, Yunting Zhang, Mengyao Li, Ya Luo, Yong Zhang, Xiaorong Wang, Haoru Tang

**Affiliations:** ^1^College of Horticulture, Sichuan Agricultural University, Chengdu, China; ^2^Institute of Pomology and Olericulture, Sichuan Agricultural University, Chengdu, China

**Keywords:** strawberry fruit, alternative splicing, isoform switch, nanopore sequencing, B-box protein 22

## Abstract

The use of alternative transcription start or termination sites (aTSS or aTTS) as well as alternative splicing (AS) produce diverse transcript isoforms, playing indispensable roles in the plant development and environmental adaptations. Despite the advances in the finding of the genome-wide alternatively spliced genes in strawberry, it remains unexplored how AS responds to the developmental cues and what relevance do these outcomes have to the gene function. In this study, we have systematically investigated the transcriptome complexity using long-read Oxford Nanopore Technologies along the four successive developmental stages. The full-length cDNA sequencing results unraveled thousands of previously unexplored transcript isoforms raised from aTSS, aTTS, and AS. The relative contributions of these three processes to the complexity of strawberry fruit transcripts were compared. The aTSS and aTTS were more abundant than the AS. Differentially expressed transcripts unraveled the key transitional role of the white fruit stage. Isoform switches of transcripts from 757 genes were observed. They were associated with protein-coding potential change and domain gain or loss as the main consequences. Those genes with switched isoforms take part in the key processes of maturation in the late stages. A case study using yeast two hybrid analysis supported the functional divergence of the two isoforms of the B-box protein 22. Our results provided a new comprehensive overview of the dynamic transcriptomic landscape during strawberry fruit development and maturation.

## Introduction

Strawberry (*Fragaria* × *ananassa* Duch.) is one of the most popular flesh fruits consumed. It is also economically important with an overall production worldwide exceeding 12 million tons in 2019 [http://www.fao.org/faostat/en/]. The fruits provide us essential nutrients such as ascorbic acid, anthocyanins, and proanthocyanidins (Rekika et al., 2005), which are believed to be health promoting. The general strawberry fruit refers to the swollen receptacle with achenes dotted on the surface. It does not have bursts of ethylene production or respiration during ripening in contrast to those climacteric fruits. Within the category of non-climacteric fruits, it has distinct characteristics compared to that of grape and raspberry fruits, regarding the fruit maturation (Fuentes et al., 2019). The allo-octoploid origin of the species also adds the complexity to our understanding of the maturation process (Edger et al., 2019; Hardigan et al., 2021). Early studies aiming to unveil the regulatory networks either from the transcriptomic, proteomic, or epigenomic level have brought us with wealth of knowledge involving the development of essential fruit qualities (Bianco et al., 2009; Estrada-Johnson et al., 2017; Sánchez-Sevilla et al., 2017; Cheng et al., 2018; Li et al., 2019b), but the detailed regulation landscape was far from clear.

The physiological and biochemical changes of the fruits during growth and maturation have been linked to the coordination of internal hormone production, sensing, and signaling, genetic regulation, as well as dynamic reprogramming according to the stage and environmental cues. Auxin and abscisic acid (ABA) are well-known central hormones for strawberry fruit development and ripening. During the initial fruit set and development, auxins, mainly synthesized in the achenes, are transmitted to the receptacle to promote the growth of the false fruit (Kang et al., 2013; Estrada-Johnson et al., 2017; Feng et al., 2019). The onset of the fruit ripening, ABA, which is antagonistic to IAA, could induce a series of ripening reactions relating to the color change, texture alteration, and flavor formation (Ji et al., 2012; Liao et al., 2018; Li et al., 2019a). Other phytohormones, including gibberellic acids (GAs) (Csukasi et al., 2011; Zhou et al., 2021), jasmonic acid (JA) (Mukkun and Singh, 2009; Garrido-Bigotes et al., 2018), as well as brassinosteroids (BRs) (Chai et al., 2013) are also involved, although their detailed functions are still being disclosed. Several key genes mediating the developmental and/or ripening changes of strawberry fruits were identified. Typical examples included the hub transcription factor SEPALLATA (FaMADS9) in controlling the flesh development and ripening (Seymour et al., 2011); the FaMYB10 factor, which transactivated the structural genes in the biosynthesis of anthocyanins (Castillejo et al., 2020); the FaGAST2, which participated in the regulation of cell expansion and then the fruit size (Moyano-Cañete et al., 2013); the FaEGS2, a key regulator for the eugenol production to the final aroma formation (Medina-Puche et al., 2015); and the recent FaRIF transcription factor, which systematically controlled the fruit ripening (Martín-Pizarro et al., 2021). Most of them were differentially transcribed upon the ripening process. This list is increasing along with the research progress.

Besides the transcription efficiency modulation, the alternative promoters and alternative polyadenylation sites also contribute to the regulation of transcript isoform production. The alternative transcription start or termination site selections (aTSSs or aTTSs) could further assist in the post-transcriptional regulation by affecting the mRNA stability and protein translation efficiency. It is evident that post-transcriptional modifications have been involved in physiological and adaptive regulations. Alternative splicing (AS) is a typical post-transcriptional regulation in defining proteomic diversity. It plays crucial roles in modulating the development process and environmental adaptations (Staiger and Brown, 2013; Szakonyi and Duque, 2018; Chen et al., 2020). In the nine selected plants, ~40–70% of the multi-exon genes underwent different pre-mRNA processing (Chamala et al., 2015). In fruit plants, including grape, apple, sweet orange, and woodland strawberry, 20–60% of all genes had various AS transcripts, several of which were consensus (Sablok et al., 2017). In the recently updated genome of *Fragaria vesca*, 10,176 genes (out of the 28,588 annotated genes) have spliced variants (Li et al., 2019c). Likewise, a total of 20,229 alternative spliced isoforms, dominated by the type of intron retention (IR) had been observed in the full-length transcriptome of the cultivated strawberry (Yuan et al., 2019). However, the allopolyploid genome of the cultivated strawberry has given rise to transcripts of high similarity from different sub-genomes, which makes it difficult to detect and accurately quantify transcripts, especially those from IR as revealed by Kuo et al. (2020).

The usage of one predominant isoform of a gene could be replaced by another one from the same gene under certain circumstances. These events are referred to as isoform switches, which have important biological implications such as the protein structure plasticity, mRNA diversity, and RNA stability (Vitting-Seerup et al., 2019; Xing et al., 2020). Despite the advancements in the finding of the genome-wide alternatively spliced genes in the strawberry, it remains unexplored to what extent the aTSS, aTTS, and AS have contributed to the complexity of transcriptome. How the diverse transcripts respond to developmental cues, and what relevance these outcomes have to the gene function remain open questions. These gaps might be ascribed not only to the lack of a reliable and complete genome assembly at the time of their studies but also to the intrinsic property of the short-read–based isoform discovery strategy.

Long-read sequencing techniques, represented by the PacBio and Nanopore platforms could result in full transcripts (cDNA or RNA), which provide us unprecedented opportunities to detect the accurate exon connectivity and isoform complexity. Without the assembly procedure, it can also accurately quantify splicing forms to detect isoform-switched transcripts (Zhu et al., 2017; Qiao et al., 2019; Cui et al., 2020). In the present study, we utilized the PromethION (Oxford Nanopore Technologies, ONT) platform to detect the genome-wide transcripts across four successive developmental stages. Furthermore, the switches of the splicing isoforms were analyzed to reveal their potential roles in the gene regulation and to explore the complexity of the transcriptome in the fruit development and ripening process.

## Materials and Methods

### Plant Materials

Fruits of the strawberry cultivar “Benihoppe” were collected from the research field of the university located in Chongzhou city of Sichuan Province, China. The plantlets were grown in late August and started to flower at the beginning of October under natural light conditions (short day, ~10 h light). Four successive developmental stages were judged on fruit skin colors: small green (SG), white (W), turning (TURN), and full red (FR) as described by Sánchez-Sevilla et al. (2017). At least 10 fruits with uniform size of the same stage were harvested as one biological sample. A total of 12 samples were collected in 2019. All samples were immediately frozen in liquid nitrogen before storing at −80°C.

### RNA Extraction and Library Preparations for Nanopore cDNA Sequencing

The total RNA was extracted from the fruits using an improved CTAB-based protocol (Leh et al., 2019). One microgram of total RNA with high purity and integrity (RIN > 8.5) was used for the library construction. A cDNA-PCR sequencing kit (SQK-PCS109) was used referring to the protocol of ONT. Briefly, full-length cDNAs were enriched by the template-switching activity of the reverse transcriptase. Specific PCR adapters were added at both ends of the first-strand cDNA. After 14 circles of PCR amplification using the LongAmp Taq (NEB, USA) enzyme, ONT adaptors were ligated to the amplified products. Afterward, Agencourt XP beads were used for DNA purification according to the manufacturer's protocol. The final 12 cDNA libraries were loaded into the FLO-MIN109 flowcells and sequenced on the PromethION platform at Biomarker Technology Company (Beijing, China).

### Base Calling and Long Reads Mapping

Base calling was performed by using the MinKNOW (v2.2, Oxford Nanopore) software on the sequencing compute module. Raw reads were classified using the cdna_classifier.py script in the pychopper package (v2.5.0) with default parameters except that reads with a length <500 nt were discarded. Only those reads that fell into the full-length category were used for further analysis. Adaptors were removed by the Porechop package (v0.2.4) with default settings. Due to the high-error–prone property of the ONT reads, we used IsONcorrect method (Sahlin et al., 2021) to correct the reads. Minimap2 was then used to map the curated reads back to the octoploid *F*. × *ananassa* genome reference (“Camarosa,” v1.0a1) with parameters “-ax splice -I 100000G –cs –MD –secondary=no –a.” Known and novel genes/transcripts identification and quantification were conducted using the TALON long-read pipeline (Dana et al., 2019) with the latest genome annotation (v1.0a2) (Liu et al., 2021). The annotation errors in the original GFF3 files (FxaC_15g00020) were manually corrected before use. Finally, the raw counts of transcripts with at least three reads in more than two samples were exported from the TALON database for the subsequent analysis.

### Differential Gene Expression and Isoform Switch Analysis

The edgeR (v3.13) package was employed for differentially expressed transcript detection, which uses the negative binomial distribution to model gene counts. Normalized expression values were calculated and presented as counts per million reads (CPM). The developmental stage was treated as a cofactor to fit the counts using the general linear model. Those transcripts with a log2 -old difference ≥2 or ≤ -2 and the Benjamini–Hochberg (BH) adjusted *p* < 0.01 were selected. Functional annotation of the isoforms was conducted through Blastp sequentially against plant proteins stored in the PLAZA4.0, NCBI RefSeq plant, and NCBI non-redundant protein sequence database (release 20210620) by using the TOA pipeline (Mora-Márquez et al., 2021). Significant isoform switch events were detected by identifying occurrences of the isoform relative abundance change (|dIF| > 0.1 and FDR < 0.05) between replicated stages in the R package IsoformSwitchAnalyzeR (v1.14.0) (Vitting-Seerup et al., 2019). The potential consequences of the aTSS, aTTS, and AS events were identified and analyzed using software, including the signalP-5.0, CPC2, IUPRED2A, and PfamScan. The existence of upstream open reading frames (uORFs) in the transcripts was predicted using the script uORF-detector.pl (https://github.com/caballero/uorf-detector/blob/master/uORF-detector.pl). For the gene set enrichment analysis (GSEA), the clusterProfiler package (v4.0.2) (Wu et al., 2021) was used with BH adjusted *p* < 0.05 as a threshold.

### RT-PCR and RT-qPCR Validation of the Selected AS Transcripts

To validate the alternatively spliced transcripts, reverse transcription PCR (RT-PCR) and real-time quantitative PCR (RT-qPCR) assays were performed. Five genes with intron retention/exon skipping isoforms were selected. Primers located on the exons flanking the skipped or retained intron(s) were designed (Supplementary Table 1). If the PCR products were difficult to be discriminated in agarose gels, primers specifically spanning the alternative splice junction to target the known (annotated in the genome reference) or the novel isoforms were designed. All primers were synthesized by Sangon (Shanghai Sangon Biological Engineering and Technological, China). A reaction system of 20 μl (1 pmol of each primer, 1 μl of cDNA template from each developmental stage, and 10 μl of 2 × PCR reaction mixture from Clontech) was established. All amplifications were done on a T100 (BioRad, USA) system with conditions: 95°C 5 min; 35 cycles of 95°C 30 s, 58°C 30 s, 72°C 30 s, and 72°C 10 min. RT-qPCR was employed to validate the differentially expressed FxaC_22g17110.t1 and TALONT000214939 in the four successive developmental stages using the same protocol as our previous report (Bai et al., 2019) with specific primers listed in Supplementary Table 1. We chose these two transcripts because they were derived from the same MADS-box protein-coding gene and had a predicted uORF consequence due to AS. The *beta-actin2* gene (LOC101313255) was used as the internal control to normalize the expression values among stages.

### Yeast Two-Hybrid Assay

It has been revealed that the B-box-containing protein (BBX) family members could physically interact with ELONGATED HYPOCOTYL5 (HY5) to fulfill their function (Lin et al., 2018). We selected the identified BBX22 gene (FxaC_12g32290) with isoform switches discovered in this study. The CrY2H-seq system (Trigg et al., 2017) was used to experimentally test the functional divergence of the two variants. The two BBX22 transcripts were cloned into the pADlox vector using a ClonExpress II one-step cloning kit (Vazyme, China) with primers listed in Supplementary Table 2. The ORF sequences of the strawberry HY5 transcript (FxaC_7g30690.t1) and the constitutive photomorphogenic 1 gene (COP1, FxaC_17g38671.t1) were cloned into the pDBlox vector using the same protocol. Fussed constructs were transformed into JM109 chemical competent cells. pAD-BBX22-lox and pDB-HY5/COP1-lox plasmids were purified using a Mag-MK plasmid DNA mini-preps kit (Sangon, China) and transformed into the yeast strain Y8800 and CRY8930, respectively, using a standard lithium acetate and polyethylene glycol method. The two strains with AD and DB were mated according to a published protocol (Trigg et al., 2017). The pDB-HY5/COP1-lox yeast strains were mated with the Y8800 yeast strains transformed with the pAD-lox empty plasmid to test the autoactivation. A final of 20 mM of 3-amino-1,2,3-triazole (3-AT) was used to suppress the existence of background transactivation of the BBX22 protein. Diploid cells were selected on the Sc-Leu-Trp media. Plates of Sc-Leu-Trp-His + 20 mM 3-AT were used to select interacting pairs. Positive clones were picked with a tooth stick and tested on selective plates Sc-Leu-Trp + 20 mM 3-AT + 1mg/L cycloheximide (CHX) to deplete *de novo* self-activations. Only those that were positive for the HIS3 reporter gene activation and negative for CHX growth were considered positive interaction pairs.

### Data Availability

The ONT full-length transcriptome data could be accessible through the CNGB nucleotide sequence archive (https://db.cngb.org/cnsa/home/) with an accession number CNP0002170. The updated gene annotation file derived from the current data are available at Figshare (https://figshare.com/articles/dataset/talon_observedOnly_gtf/19358903/1).

## Results

### Overview of the Sequencing Data

**Figure 1:**
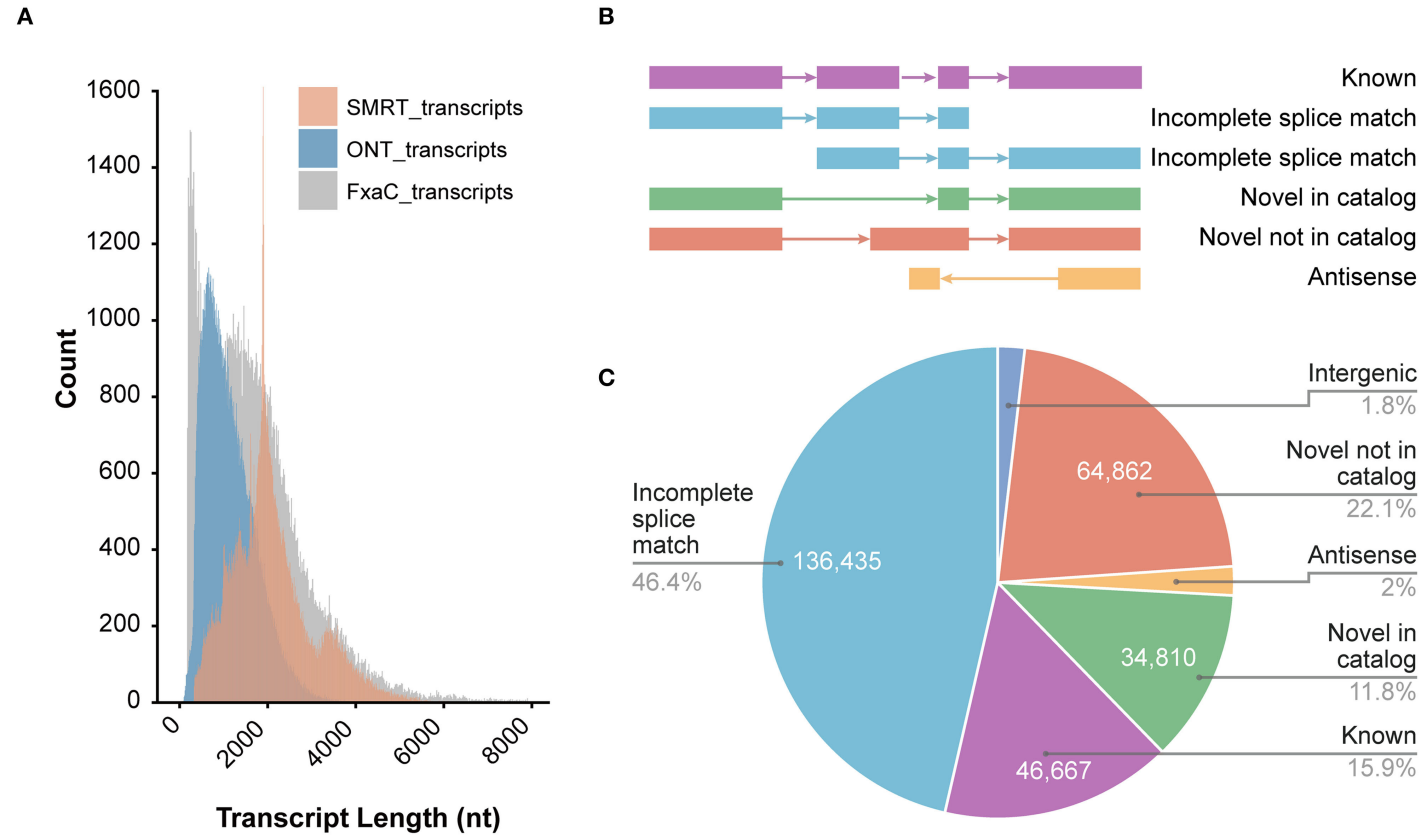
Overview of the ONT sequencing data. **(A)** The length distribution of ONT transcripts in comparison with SMRT and those known transcripts in the genome annotation. SMRT_transcripts were from the non-redundant transcripts in the PacBio sequencing project of the previous study (SRA record PRJNA510532) using the same cultivar. FxaC_transcripts were extracted from the latest genome annotation (v1.0a2) profile. **(B)** The known or novelty definition diagram according to the category of TALON pipeline. **(C)** Counts of the transcripts and the proportion of each category.

We obtained a total of 52,665,449 raw ONT long reads from strawberry fruits with an average length of 815 nt. The maximum length of the reads reached 12,295 nt (Supplementary Table 3). In all samples tested, full-length transcripts (with primers existing at both ends of reads) accounted for ~88% of all reads. After removing the 5,917,277 truncated or too short reads, 12 libraries generated 46,748,172 reads with an average read quality of 11.3 (Table 1). The read length distribution is shown in Figure 1A. All these reads were put into the IsONcorrect pipeline for base corrections. Meanwhile, we also included the PacBio SMRT long reads generated from the same cultivar (SRA record PRJNA510532) for comparison. We only investigated the read length distribution since only the error-corrected and redundance-removed consensus transcripts were available. The average length of SMRT reads reached 2,100 nt, which was much larger than that of the ONT transcripts (Figure 1A). Additionally, this metric was strikingly different from that of the transcripts in the updated genome annotation profile (v1.0a2, FxaC_transcripts), in which many transcripts with lengths <500 nt were labeled (Figure 1A).

**Table 1:** Full-length non-chimeric transcripts of the Oxford nanopore sequencing datasets.

	**Small green^a^**	**White**	**Turning**	**Full red**
Reads number	10,051,859	10,614,343	13,509,638	12,572,332
Average length	711	723	711	761
Median length	644	646	643	677
N50 length	705	748	744	799
Max read length	7,437	6,999	6,603	7,214
Average read quality	11.1	11.3	11.3	11.3

a *Calculated from three biological replicates*.

After mapping all curated reads to the cultivated strawberry genome, the TALON pipeline was employed (Dana et al., 2019). Known genes, transcripts, and splicing patterns defined in the genome annotation GTF file were initially built around an SQLite database. By using the TALON program, the mapped long reads were clustered and assigned to known or novel genes/transcripts by comparing with the existing models. The novelty of the models was classified into either known, incomplete splice match, novel in catalog (NIC), novel not in catalog (NNC), or antisense (Figure 1B). Of all 293,990 clustered transcripts, 46% fell into the incomplete splice match category. Another 46,667 known transcripts were recovered. A total of 110,888 (account for 37.7%) transcripts were newly discovered in this study (Figure 1C), including 64,862 transcripts (58.5%) derived from unreported splicing patterns. These results highlighted the powerful resolution of the ONT techniques in revealing the complete landscape of transcriptomes in strawberry plants.

### Differentially Expressed Transcripts Along the Fruit Development Process

Assignment of each long read to a particular gene locus makes it possible to quantify gene/transcript expressions by simply counting the number of each isoform without assembly. This expression value was normalized into counts per million (CPM). An initial quality assessment indicated a higher correlation coefficient (Pearson's) among the three replicates of the same stage than that between different stages using all transcripts' expression values (Supplementary Figure 1). Previously, pioneer investigations had been conducted to reveal the transcriptomic changes either using the array-based or second-generation high throughput (HT) sequencing techniques in wild *Fragaria* species or cultivated strawberry plants (Kang et al., 2013; Medina-Puche et al., 2016; Estrada-Johnson et al., 2017; Hu et al., 2018; Li et al., 2019b, 2021). Here, those genes which have a minimum CPM of one in at least two samples were deemed to be truly expressed. We observed 98,160 transcripts derived from 41,032 genes expressed in the four successive developmental fruit stages (Supplementary Table 4). This number was slightly lower than what was reported by using short-read–based methods (Hu et al., 2018).

**Figure 2:**
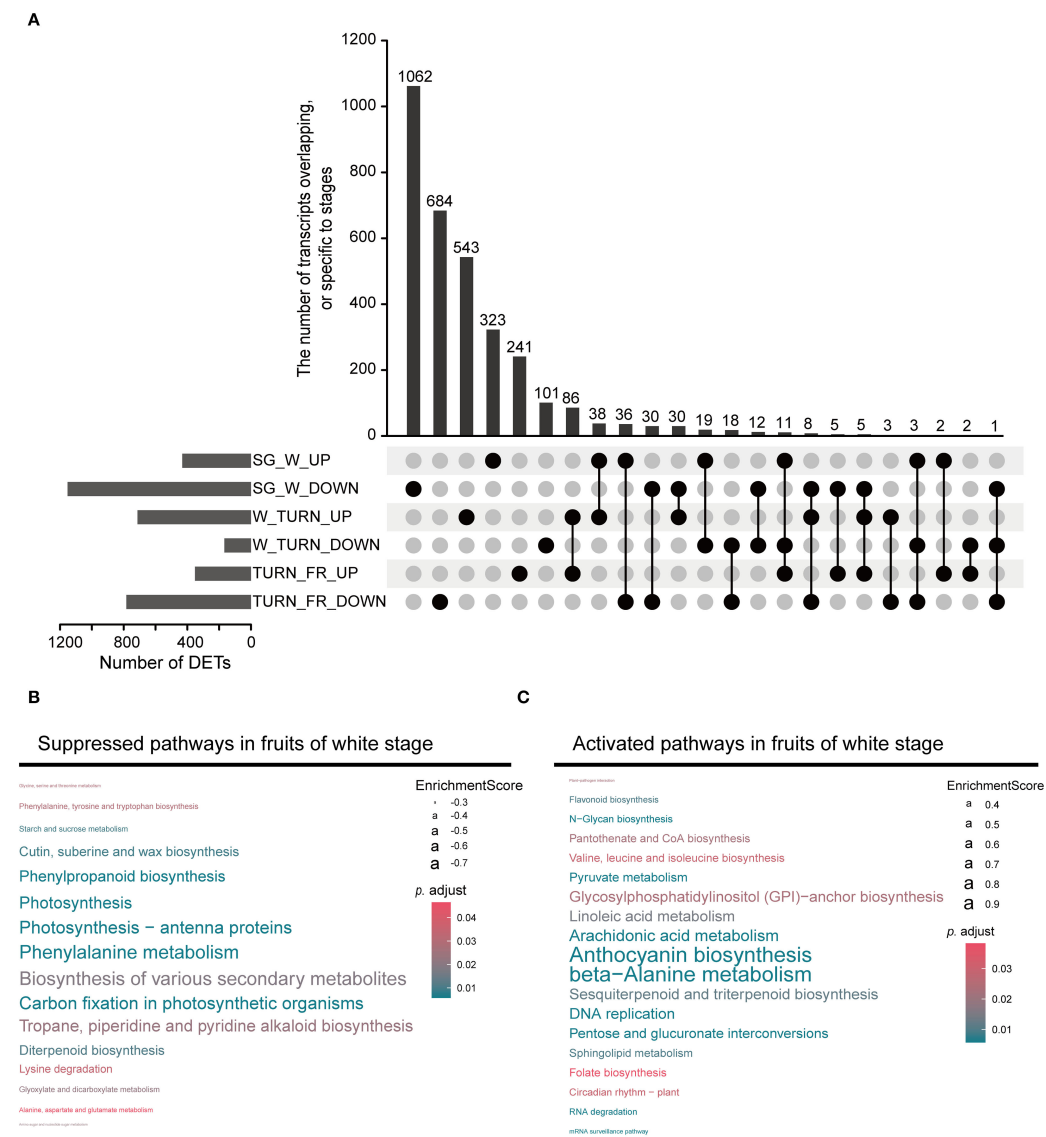
The differentially expressed transcripts (DETs) detected between the two neighboring stages and the enriched gene set. **(A)** The upset plot representing the DETs in comparison between the small green stage (SG) and the white stage (W), the white stage (W) and the turning stage (TURN), and the turning stage (TURN) and the full red stage fruits (FR). **(B)** Gene set enrichment analysis (GSEA) revealed the suppressed pathways in the white stage fruits. **(C)** The activated pathways in fruits of white stage detected by GSEA.

Through pairwise comparison between samples of adjacent stags during maturation (SG *vs*. W, W *vs*. TURN, TURN *vs*. FR), we detected a total of 3,600 non-redundant transcripts, which were differentially expressed in at least one comparison using the defined criteria. The largest number of DETs were observed in the comparison of the white fruit and small green fruit stage, with 432 being up-regulated and 1,152 being down-regulated (Figure 2A). This result implied that fruits at the white developmental stage underwent the most striking transcriptional regulatory changes. GSEA of the DETs presented us with more details of the functional pathways being controlled. Several ripening-related pathways had already been altered at this time point. For example, the most significantly suppressed pathways included photosynthesis-related, biosynthesis of various secondary metabolites, phenylpropanoid biosynthesis pathway, tropane, piperidine, and pyridine alkaloid biosynthesis, and diterpenoid biosynthesis (Figure 2B and Supplementary Table 5). In contrast, the anthocyanin biosynthesis pathway was activated at this stage, although this pigment started to accumulate later till the turning stages. Moreover, pathways including beta-alanine metabolism, arachidonic acid metabolism, linoleic acid metabolism, pyruvate metabolism, pentose and glucuronate interconversions, folate biosynthesis, and sphingolipid metabolism were activated (Figure 2C). These pathways could directly impact the final nutrient quality of the fruits. All these results pinpointed the critical transition of fruit growth to ripening in the white fruit stage.

When anthocyanins started to accumulate at the turning stage, the corresponding biosynthetic pathway continued to be up-regulated (Supplementary Table 6). More salient features were observed about the amino acid metabolism at this time point. Beta-alanine metabolism, phenylalanine metabolism, lysine degradation, phenylalanine, tyrosine and tryptophan biosynthesis, and valine, leucine, and isoleucine biosynthesis were activated. In contrast, arginine biosynthesis, cysteine and methionine metabolism, and histidine metabolism were suppressed (Supplementary Table 6). Other boosted pathways at this stage were associated with the carotenoid biosynthesis, starch and sucrose metabolism, and fatty acid degradation.

In the transition process to the full red stage, several lipid/fatty acid metabolism pathways, including sphingolipid metabolism, fatty acid biosynthesis, fatty acid degradation, and glycerolipid metabolism were enhanced (Supplementary Table 7). The amino acid metabolism-related pathways were still differentially controlled in this process.

### aTTS and aTSS Occurred More Common Than AS in the Strawberry Fruit Development

**Figure 3:**
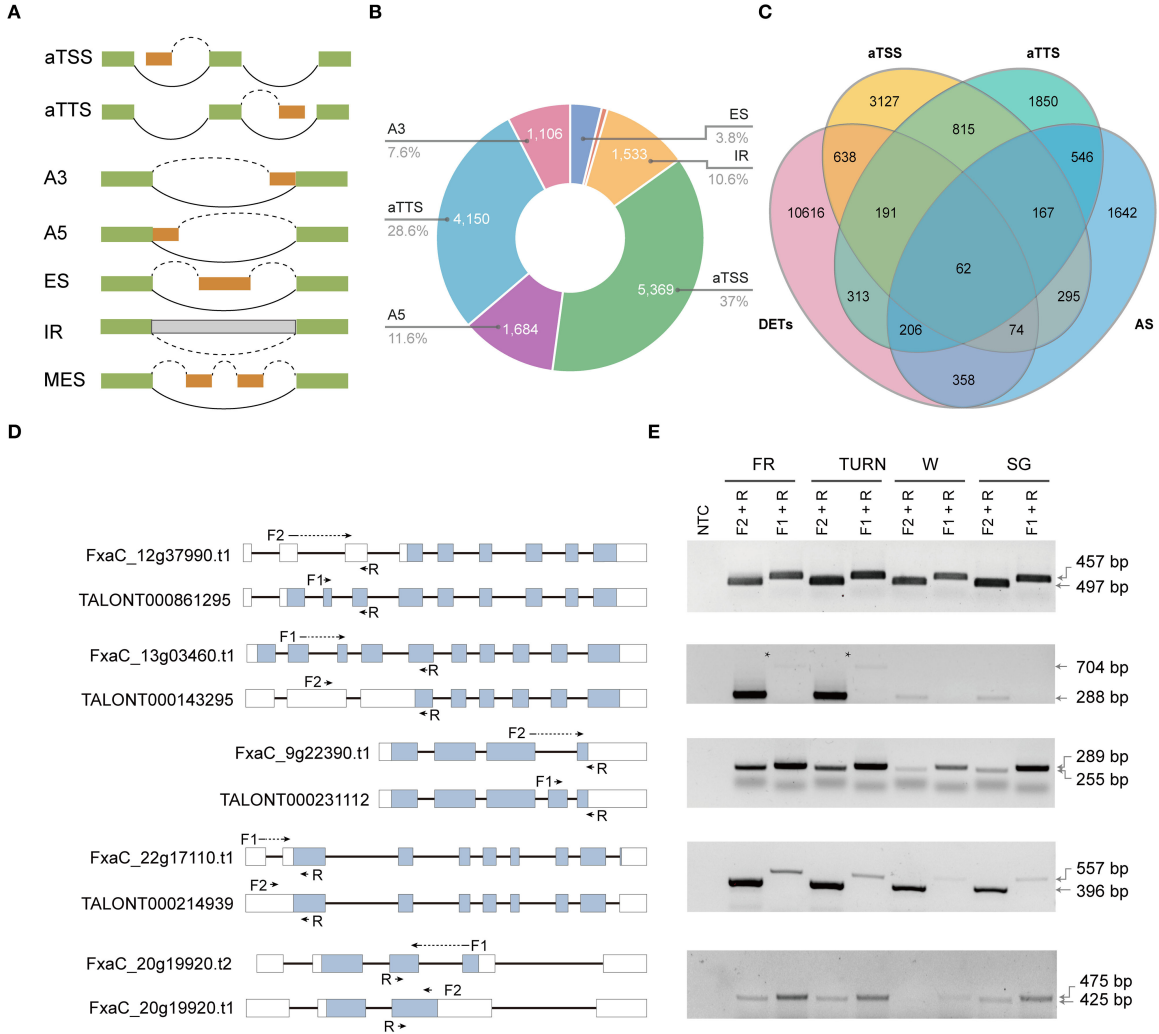
Alternative isoform production events (aTSS, aTTS, and AS) investigated and their relative contribution to the transcriptome complexity and transcript expression. **(A)** The isoform production patterns defined. **(B)** The number and proportion of transcripts derived from the seven modes. **(C)** Venn diagram showing the overlapping transcripts derived from aTSS, aTTS, or AS with those being differentially expressed. **(D)** The gene model of the selected genes each with two selected isoforms. The location of the primers was labeled using arrows. Those primers spanning an intron were marked using dotted lines. **(E)** RT-PCR validation of the isoforms with specific primer combinations in the four successive developmental stages.

Alternative promoter selection, alternative termination site, and selectively splicing of introns corporately contribute to the diversity of isoforms. They also exert major mechanisms of gene expression regulation (Staiger and Brown, 2013; Policastro and Zentner, 2021). The genome-wide splice junction selection has been investigated in strawberry plants recently using either Illumina or PacBio reads (Hu et al., 2018; Yuan et al., 2019). But the relative contribution and the functional effect of these molecular processes have been rarely analyzed. We could address these questions by using the full-length transcriptome data since the full-length cDNAs were derived from template-switching reverse transcriptions, and the transcripts were directly sequenced without fragmentation. In this study, seven types of isoform production events including aTSS, aTTS, and the five major types of AS (Figure 3A) were assessed. Of the 14,497 isoform regulation events detected in 10,283 genes, aTSS and aTTS were the dominant events, accounting for 37.0 and 28.6%, respectively. AS contributed to ~34.0% of the multi-isoform transcripts. Among the detected AS categories, the number of alternative donor (A5) events (11.6%) was slightly higher than that of intron retention (10.6%), which was found to be the most prevalent splicing type in previous reports (e.g., Hu et al., 2018). Interestingly, 1,235 isoforms were generated through both aTSS and aTTS processes (Figure 3C). Among these isoforms, 229 were also coupled with AS.

The aTSS and aTTS are involved in gene regulation by modulating both the stability and translation of mRNAs, while AS is directly related to the relative abundance of a specific transcript. We also investigated to what extent was the isoform generating events related to the transcript expression. Before analysis, we extended our DET analysis to include all pairwise comparisons of the four developmental stages. Finally, a total of 12,458 transcripts were selected as differentially expressed based on the defined criteria. Among these DETs, only 700 (5.6%) were overlapped with the transcripts derived from AS (Figure 3C). In contrast, 965 isoforms (7.7%) derived from aTSS were differentially expressed. Similarly, 772 aTTS event products (6.2%) were also found in the DET list. To validate the detected AS products, five genes were picked. The two transcripts of each gene were analyzed using RT-PCR with transcript-specific primers in fruits of all four developmental stages (Figures 3D,E, and Supplementary Figure 4).

### Isoform Switching and Their Functional Implications in the Fruit Development of Strawberry

**Figure 4:**
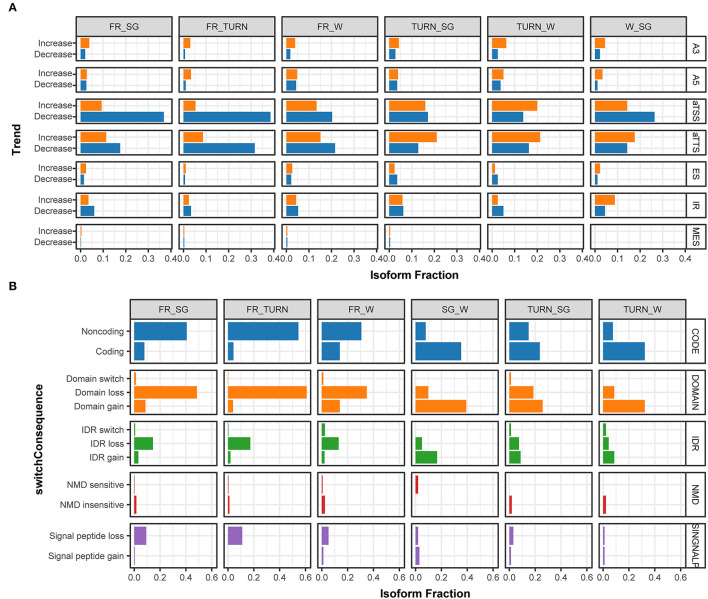
The fraction of isoforms with significant usage switching associated to aTSS, aTTS, and AS events along with the fruit development and the predicted functional consequences. **(A)** The fraction of isoforms that significantly changed their usage (increasing, colored in orange; and decreasing, colored with light blue) in the pairwise comparison among the four stages. Isoforms derived from alternative 3' acceptor site (A3), alternative 5' donor site (A5), alternative transcription start site (aTSS), alternative transcription termination site (aTTS), exon skipping (ES), intron retention (IR), and multiple exon skipping (MES) were summarized separately in the IsoformSwitchAnalyzeR package. **(B)** The percentage of isoforms associated with functional changes ascribed to isoform switching events in the pairwise comparison of expression among the investigated developmental stages. The functional consequences of isoform switches were based on the annotation of coding potential (CODE, predicted by using CPC2), protein domain change (DOMAIN, predicted by PfamScan), intrinsically disordered regions (IDR, *via* IUPred2A), sensitivity to Non-sense Mediated Decay (NMD, when harboring premature termination codons), and signal peptide (SINGNALP).

To go further deep into the complex transcripts, the relative abundance of each transcript of the same gene (isoform fraction, IF) and the alternation of the fraction (dIF) were statically tested by pairwise comparison throughout the development. We detected 1,411 transcripts associated with 880 significant isoform switch events in 757 genes (Supplementary Table 8). This transcript usage transition could indirectly reflect the promoter preference and the splicing junction selection. Therefore, isoform production events of these switching genes were further analyzed. During the fruit development process, it was obvious that some of these events were not equally used (Figure 4A). For example, when compared with the fruits of the white stage, the turning stage fruits (TURN *vs*. W) used aTSS to produce higher proportions of isoforms with increasing IF. In contrast, the full-red fruits (FR *vs*. W) had a higher fraction of aTSS transcripts declining their usage (Figure 4A). Again, an opposite preference for aTTS was observed in the turning-stage and full-red stage fruits when compared with other developmental stages. Intron retention events are of particular interest because they dramatically change isoforms. This type of AS was enhanced in fruits of the ripening stage (TURN and FR) but repressed in fruits before the white stage, although it was not significantly enriched by using Fisher's exact tests (Supplementary Figure 2).

The potential consequences of the identified isoform switch events due to aTSS, aTTS, or AS could be predicted by integrating the information of the products and their functional annotations. We paid close attention to those that have a clear functional impact including protein-coding potential, conserved domains, signal peptides, intrinsically disordered regions (IDR), and mRNA sensitivity to non-sense mediated decay (NMD). The largest proportions of genes with isoform switches were with functional changes involved in the domain gain/loss, followed by the protein-coding potential (Figure 4B). Specifically, in full red fruits, a large proportion of transcripts (~50%) were predicted to encode proteins missing one or more domains. On the contrary, the fraction of transcripts with domain gain was produced in the fruits of the turning stage. Similar trends were observed for the protein-coding potential and IDR gain/loss events due to isoform switches. In total, 36 transcripts were classed as NMD insensitive while only eight were predicted to be sensitive to NMD according to the 50-nt rule (Lindeboom et al., 2016). Signal peptide losses were increasingly preferred along with the growth of fruits (Figure 4B). These results indicated that isoform switches during the fruit development had an immense impact on the protein function rather than RNA stability.

**Figure 5:**
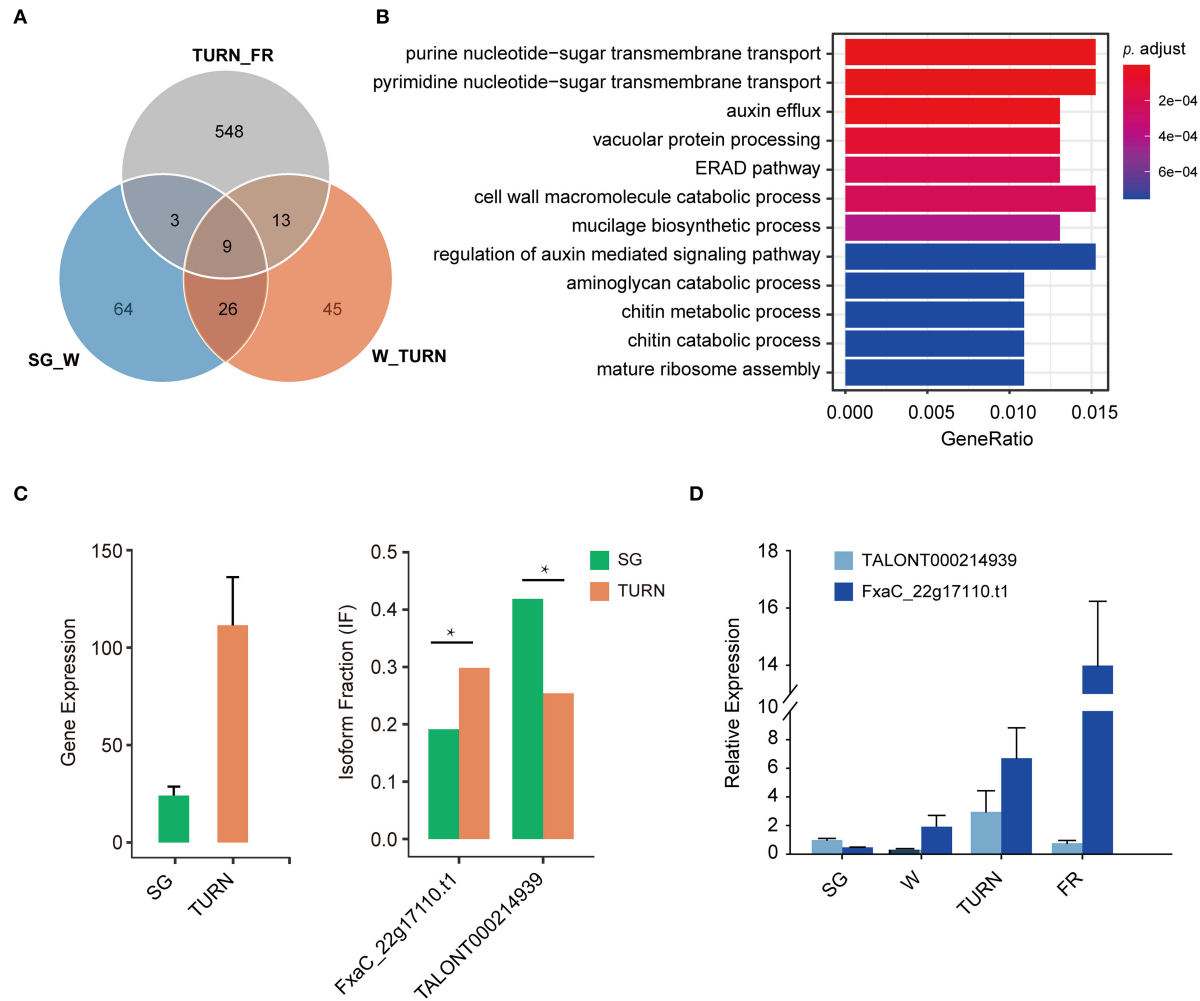
The number of genes with isoform switch events and the enriched GO terms of these genes. **(A)** Isoform switch events detected in the adjacent fruit developmental stages, **(B)** the corresponding enriched GO term associated with the switching genes in the comparison of TURN *vs*. FR, **(C)** an example of isoform usage alternation between the two representative isoforms of the gene FxaC_22g17110, and **(D)** RT-qPCR validation of the expression level of these two transcripts in strawberry fruits at four developmental stages. SG, small green; W, white fruit; TURN, turning stage; FR, full red stage. Significance levels were estimated by two-sided Fisher exact tests, * indicated FDR corrected *p* < 0.05.

To further answer the question that which biological pathway suffered much from these isoform switches, GSEA was carried out. We focused only on the comparisons SG_W, W_TURN, and TURN_FR, because they were meaningful in a biologic context. Among these pairs, as shown in Figure 5A, a much greater number of transcripts derived from switch events were observed in the comparison of the turning and full red stage. Only 22 and 12 transcripts also suffered from usages alteration in the other two comparing pairs, respectively. GO term enrichment analysis of these genes in the TURN_FR group demonstrated that several biological networks were significantly affected. The top 12 were three terms related to protein synthesis and degradation, four terms associated with cell wall structural compounds metabolism, two terms involved in auxin transport and signaling, and three terms linked to sugar transportation and metabolism (Figure 5B). It has been previously reported that the splicing factors themselves were differentially spliced (Hartmann et al., 2018). We observed that three serine/arginine-rich splicing factors and two splicing factor subunit encoding genes (FxaC_20g19920, FxaC_28g05430, FxaC_24020, FxaC_10g02450, FxaC_27g45580) had undergone isoform switches. One example was illustrated in Supplementary Figure 3. FxaC_20g19920 encodes a serine/arginine-rich splicing factor protein. The retention of the third intron of the gene produced a transcript with an unusually long 3' UTR when compared with the canonical form. Also, it introduced a premature termination codon (PTC) for protein translation, which is a typical sign for NMD-mediated degradation (Supplementary Figure 3A). Moreover, we also observed isoforms with short introns being retained in the 5' UTR, which was difficult to be detected using conventional HT sequencing methods. Take one gene, for instance, the FxaC_22g17110, which encodes an MADS-box transcription factor, produced 18 isoforms (Supplementary Table 4). The transcript with a 161 nt intron being reserved (TALONT000214939) had a 438 bp 5' UTR region, in which two short uORFs were found (Supplementary Figures 3B,C). The gene itself increased expression when comparing the level in small green stage to the turning stage (Figure 5C). Regarding the isoform changes, of all the isoforms generated from the gene, the longer one (TALONT000214939) significantly dominated the isoform types in small green fruits, while the shorter one (FxaC_22g17110.t1) increased its fraction in fruits of the turning stage (Figure 5C). The last 16 isoforms remained relatively stable in these investigated stages. The fluctuations of the two isoforms were confirmed by RT-qPCR (Figure 5D). To sum up, our results demonstrated that the alternative promoter usage, splicing of RNAs, alternative polyadenylation, and isoform switches with functional implications such as protein domain loss/gain or mRNA coding potential changes, were important regulation mechanisms for the strawberry, particularly in the late fruit developmental stages.

### The Effect of Alternative Splicing on Protein–Protein Interactions of BBX22: A Case Study

**Figure 6:**
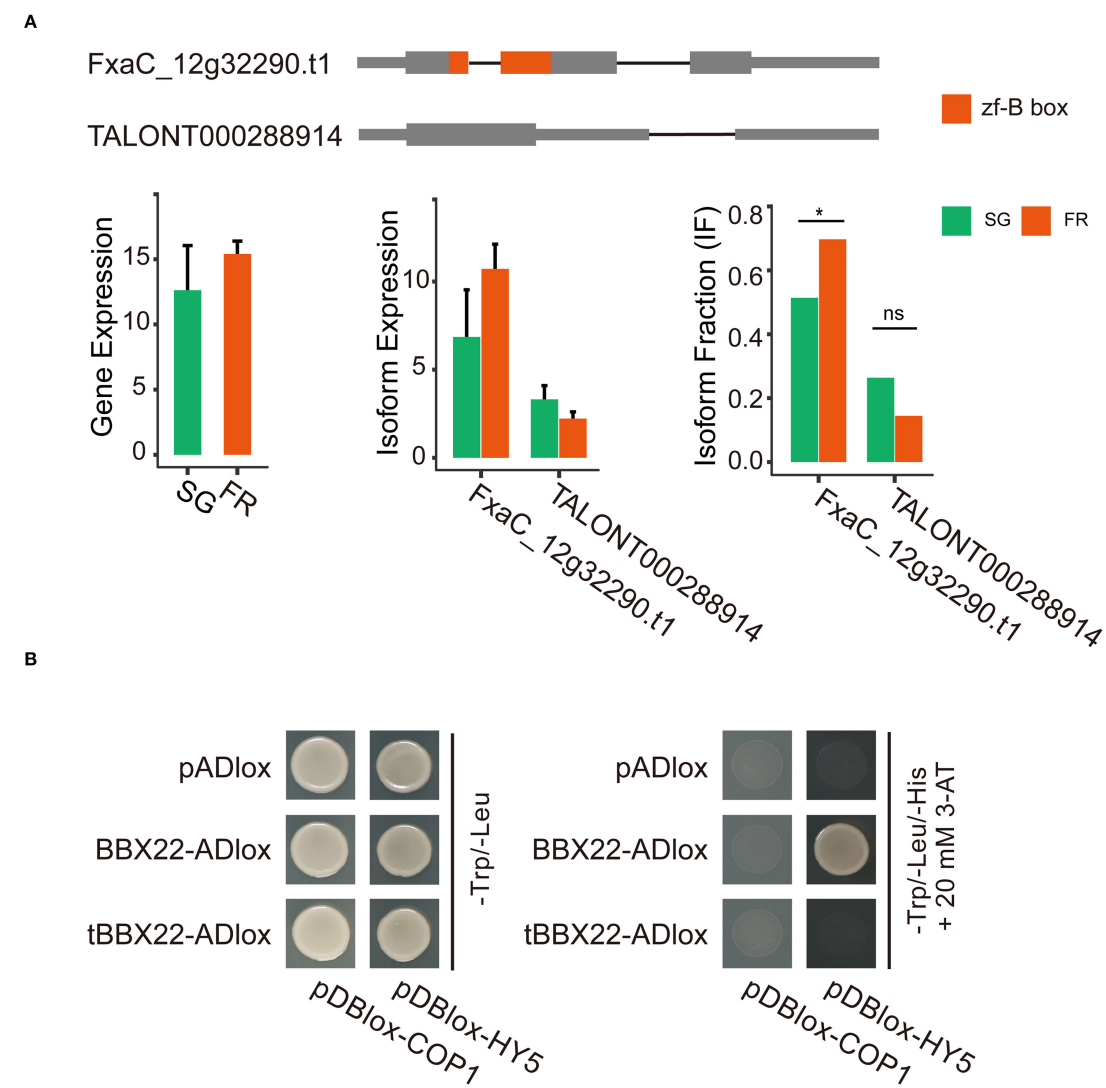
Isoform switching of the two representative isoforms of the strawberry BBX22 gene and their functional divergence revealed by yeast two-hybrid assays. **(A)** Gene models of each isoform and the abundance of the gene or the expression of each isoform in the small green and full red stage fruits. The zf-B box domain coding region is in red. Significant isoform switching events detected by DEXseq (*p*_adj_ < 0.05) implemented in the IsoformSwitchAnalyzeR package were labeled with a star (*) symbol. **(B)** Yeast two-hybrid screening for interactions between the HY5/COP1 and the BBX22 or the truncated BBX22 protein (tBBX22).

BBX proteins take part in light signaling either through transactivating transcription of the *HY5* gene or by modulating the activity of the HY5 protein. The latter route requires the protein–protein interactions. This process might be controlled by the COP1-mediated degradation *via* the 26S proteasome system (Lin et al., 2018). In the isoform switch analysis, we detected that the two transcripts (FxaC_12g32290.t1 and TALONT000288914) out of the five isoforms of the same strawberry BBX22 gene had significant isoform switch events. Both transcripts were detected in fruits of all developmental stages. The abundance of the transcript FxaC_12g32290.t1 was relatively higher (Figure 6A). Although the gene expression level was relatively stable, the isoform FxaC_12g322290.t1 increased its dominance in the red fruits, accompanied by the fraction decrease of TALONT000288914 at the same stage (Figure 6A). The two transcript variants were cloned and validated by sequencing. The deduced protein of TALONT000288914 lost its B-box-type zinc finger domain (zf-B) due to the production of a premature stop codon in the retained 89 bp intron. It has been revealed that the zf-B box domain was required for the protein interaction with HY5 (Lin et al., 2018). We tested the physical interactions of the strawberry HY5 and COP1, with the proteins of the two distinct isoforms. Results of the yeast two-hybrid assay demonstrated that the canonical BBX22 indeed interacted with the HY5 protein. In contrast, it did not associate with the COP1. The truncated BBX22 was not able to interact with any of these two proteins (Figure 6B). Given that the Arabidopsis BBX28 protein could repress the transactivation activity of HY5 through protein interactions (Lin et al., 2018), these two AS variants of strawberry BBX22 would have distinct biological functions.

## Discussion

### Robustness of ONT in the Discovery of Full Transcripts

To systematically unravel the transcriptomic complexity, massive throughput sequencing-based analysis is becoming a cost-effective and worthwhile method. The second-generation sequencing techniques represented by the Illumina platform have been used for transcript identification, quantification, and AS pattern discovery for hundreds of plant species, including the strawberry (Kang et al., 2013; Sánchez-Sevilla et al., 2017; Hu et al., 2018; Li et al., 2021). It is superior to other methods for its high accuracy and low cost but limited by its short-read length. Long-read sequencing platforms, including PacBio and ONT, dramatically increased the read length. The avoidance of assembling of reads could deplete the errors that are brought when rebuilding the transcripts using short reads. These new techniques have been used to obtain the accurate and complete landscape of DNA molecules and RNA transcription, hence facilitating genome assembly and annotation (Li et al., 2017; Edger et al., 2019; Yuan et al., 2019). The SMRT reads of strawberry have greatly replenished the completeness of the genome information when implemented in the latest annotation (Liu et al., 2021). In this study, the ONT platform was used for the first time to test its application effect in transcript identification and quantification in strawberry. Similar to the benchmark evaluation of reads from ONT and PacBio in Arabidopsis (Cui et al., 2020), the mean length of ONT reads was shorter, but the full-length ratio and the mapping rates were higher. Our data also showed that the ONT dataset contained a wealth of unexplored transcriptomic information, especially the aTSS, aTTS, and AS usage that was not revealed by Illumina or PacBio reads earlier (Figure 1C). Given the comparatively higher throughput and lower cost than the PacBio sequencing, it is an attractive approach to use ONT-seq in detecting the complexity and dynamic changes of isoform generation events under various conditions.

### Transcriptional Transition in the White Fruit Stage of Strawberry

Our results highlighted the important transitional role of the white stage in the strawberry fruit development. Previously, significant changes in both primary and secondary metabolites in strawberry fruits were systematically studied (Fait et al., 2008; Li et al., 2019b). The photosynthetic process was suppressed throughout fruit development from our results, compatible with reports of Hu et al. (2018). Fruit photosynthesis of strawberry does exist but is unique for its low stomata density, different chlorophyll content, and chlorophyll a:b ratio (Blanke, 2002). Genes involved in the photosynthesis system, including the antenna proteins (FxaC_24g47220.t1, FxaC_23g31370.t1, FxaC_21g56590.t1, Supplementary Table 5), and genes coding the proteins in the photosystem I and photosystem II, were significantly deactivated.

The transcriptional transition has been observed for the biosynthesis of various secondary metabolites. The genes coding for the phenylcoumaran benzylic ether reductase (PCBER) were enriched in the suppressed pathways. In Poplar, this enzyme could reduce phenylpropanoid dimers to form benzyl-reduced form of neolignan G(8-5)G, which serves as an antioxidant in the xylem (Niculaes et al., 2014). The function of this enzyme in strawberry still needs to be clarified. The other type of deactivated metabolite pathway was associated with the tropane, piperidine, and pyridine alkaloid synthesis. This pathway consists of several known reactions sourced from phenylalanine, lysine, arginine, and proline metabolism. These amino acids either continuously decline or exhibit a double sigmoid behavior, with a trough in the white stage (Fait et al., 2008). These changes in result could limit the synthesis of those alkaloid compounds. In contrast, anthocyanin biosynthesis and flavonoid biosynthesis were enhanced at this stage (Figure 2C). Although the red pigments of strawberry, mainly composed of the glycosylated pelargonidins and cyanidins, start to accumulate visually in the turning stage, UDP-glucosyl transferases (Supplementary Table 5) which act as the last step of the anthocyanin biosynthesis had already been activated. Meanwhile, other anthocyanin-related genes such as those coding for the flavanone 3-hydroxylase (F3H), chalcone synthases (CHS), and dihydroflavonol 4-reductase (DFR) were also boosted in transcription. Interestingly, we observed that the diterpenoid biosynthesis pathway leading to GAs was downregulated, while the sesquiterpenoid and triterpenoid biosynthesis network was activated in the white fruit (Figure 2C). It has been recorded that in the wild strawberry plants, various GAs were accumulated in the early fruit stage but gradually decreased during maturation (Liao et al., 2018). Cooperated with auxins, GAs exerted unneglected roles in controlling fruit growth and shape (Liao et al., 2018; Zhou et al., 2021). In the cultivated strawberry, saponins (glycosylated triterpenoids) were peaked in the middle phase of fruit development, while glycosylated sesquiterpenoids accumulated predominantly in the turning and red fruit stage (Fait et al., 2008). The characterized protein involved in the biosynthesis of these products in plants is the Nerolidol Synthase1 (NES1) (Aharoni et al., 2004). In total, 21 coding transcripts for this enzyme were identified and several of them were upregulated in the white fruit stage when compared with the green stage (Supplementary Table 5). Moreover, one transcript coding for the casbene synthase, and two for pinene synthases were discovered. These terpenoids were important biomolecules for responding to fungal diseases in strawberry (Mehmood et al., 2021). Since strawberry fruits are susceptible to several fungal and bacterial pathogens, the increase in synthesis of these compounds could enhance the fruit's defenses against diversities of biological stresses in the later stages. To support this assumption, the synthesis of arachidonic acid, a potent elicitor for programmed cell death and defense response (Savchenko et al., 2010) was enhanced.

Another outstanding change observed was the production of pyruvate and its derivatives in this middle phase of fruit development. One of the most significantly boosted pathways was the beta-alanine metabolism path, in which several malonyl-CoA decarboxylase coding transcripts were activated. The enzyme could catalyze the synthesis of acetyl-CoA from malonate in bacteroids and plants (An and Kim, 1998). Moreover, the NADP-dependent malic enzymes in the pyruvate metabolism pathway were actively transcribed, leading to the production of pyruvate from malate acids. The pantothenate and CoA biosynthesis pathway was also enhanced during this stage. It has been observed that most TCA cycle intermediate products do not fluctuate much during the fruit development (Fait et al., 2008). These results prompted us to hypothesize that acetyl-CoA was particularly accumulated, on one hand, providing energies for the fast fruit growth, on the other hand, and more possibly, preparing the precursors for fatty acid synthesis. The hypothesis was in agreement with the reported accumulation of fatty acids in the white receptacle (Fait et al., 2008). The omega-6-fatty acid (linoleic acid) and sphingolipid were two examples of the terminal fatty acid derivatives, the metabolism pathway of which were all activated in the white fruit stage (Figure 2C).

### aTSS, aTTS, and AS Are Indispensable Regulation Approaches in Strawberry Fruit Development

Traditionally, AS was commonly classified into types of intron retention, exon skipping, alternative donor, and alternative acceptor selection. The occurrence of these changes could impact the fate of gene products. Alternative transcription initiation and alternative transcription termination are also molecular features that contribute to novel transcripts in plants. In this study, these two types of RNA processes were combined with the traditional four AS for convenience. Full-length cDNA long reads sequencing enabled us to detect 14,497 alternative isoform production events in 10,283 genes. Intriguingly, aTSS and aTTS in total accounted for more than 65% of the identified events (Figure 3B). This result was consistent with the observations in human genes (Reyes and Huber, 2018) and similar to the observed Arabidopsis genes' response to developmental or environmental conditions (Tomas et al., 2020). By a combination of both short- and long-read data, it has been demonstrated that in Arabidopsis, exogenously ABA treatments could significantly affect the isoform-generating patterns, particularly alternative first exon (also known as aTSS) and alternative last exon (also known as aTTS) (Zhu et al., 2017). It is not known whether these observed changes are ABA-responsively specific. In strawberry, ABA was indeed the central hormone that initiated fruit ripening (Symons et al., 2012; Liao et al., 2018; Li et al., 2019a). Several genes of ABA biosynthesis and signaling also underwent AS impact. For example, the OsABI5, encoding a bZIP transcription factor, generates two variants that exert overlapping and distinct functions in ABA signaling (Zou et al., 2007). It deserves notification that those newly produced isoforms due to ABA application in Arabidopsis were dominated by coding forms when compared with transcripts from the water-mock plants (Zhu et al., 2017). Conversely, in strawberry, in this study, noncoding isoforms were enriched in the full red fruits in comparison with fruits of other stages (Figure 4B). A large proportion of these newly identified features (519 out of 818) were not able to be functionally annotated by homology searches (Supplementary Table 8). There might be an organism- or developmental stage-specific mechanism. Developmentally regulated splice-junction selection had been observed in thousands of *Zea mays*' genes and had obvious tissue specificity (Thatcher et al., 2016). Almost half of these developmental isoform changes were derived from aTSS and aTTS. In our study, the aTSS, aTTS, and AS-derived transcripts each had a small overlap with those differentially expressed, indicating that the involvement of these biological processes may be as important as activating or repressing gene expression in strawberry fruits. Overall, these results implied that aTSS, aTTS, and AS had brought very large impact on the transcriptome complexity.

### Isoform Switches Were Directly Related to the Protein Domain Shift or mRNA Coding Ability Changes in the Fruit Ripening Regulation Networks

The usage of each transcript produced through aTSS, aTTS, and AS from the same gene could vary from condition to condition. These isoform switches of genes with immense functional consequences have been extensively documented in both animals and plants. The two splice variants of the Arabidopsis FLOWERING LOCUS M (FLM) work antagonistically with each other to finetuning the flowering process (Posé et al., 2013). In the wild strawberry, a remarkable reduction of IR events and a significant upregulation of alternative acceptor site were reported in fruits of post-fertilization. Several of these isoforms were characterized to have protein domain gain or loss effects (Li et al., 2017). We have identified 880 significant isoform switch events for 757 genes in the cultivated strawberry in this study. Most of the switch events occur in the late developmental stages, covering specific key maturation and senescence processes (Figure 5B), which represented the most striking physiological changes during fruit ripening (Moya-León et al., 2019). The corresponding isoform usage modes clearly showed that aTSS and aTTS were differentially used (Figure 4A). aTSS could alter the N-termini of proteins or bring uORF structures in the 5' UTR, which could respond to various environmental and internal cues such as light, sugar availability, and polyamine levels (Jorgensen and Dorantes-Acosta, 2012). Only a handful of these genes were functionally illustrated. The FaxC_22g17110 produced two uORFs containing transcript due to the intron retention in the 5'UTR (Supplementary Figure 3C). It might be regulated by the same protein translational machinery, or it might be subjected to NMD, as proposed by Lindeboom et al. (2016). Further proteomic or experimental data were needed to clarify its function in fruit development. Conversely, aTTS generates isoforms with different 3' ends, constituting another regulatory layer for transcript diversity. Transcripts from aTTS or IR with a long 3'UTR and a PTC feature might be amenable to NMD before translation (Kalyna et al., 2012). In plants, from the results of very few studied model plants, such as in *Zea mays*, nearly half of the isoform switch events were associated with NMD (Thatcher et al., 2016). In contrast, we only observed 44 (out of 1411) transcripts with predicted NMD sensitivity changes. One example was the splicing factor coding gene FxaC_20g19920, which augmented its NMD-sensitive form in the ripening stage (Supplementary Figure 3A). This result indicated that the RNA abundance and protein translation modulation *via* NMD of mRNA was not the major regulatory mechanism here. But given the evidence that splicing factors (SFs) have extensively participated in the spliceosome assembly and have undergone both AS and isoform switches themselves, the NMD of spliced isoforms of these SFs could still impact the overall transcription profiles. ES or IR are two mostly studied AS types that have obvious functional consequences including protein domain gain or loss, protein structure interference (e.g., IDR), and introduction of PTCs. Besides the influence in the mRNA coding property discussed above, protein domain loss or gain through aTSS, aTTS, and AS dominated the proteomic diversity regulation throughout the ripening process (Figure 4B). This highly consistent pattern is particularly noteworthy in the late developmental stages. As expected, the BBX22 protein coding by the truncated isoform due to IR events compromised its ability in interacting with the HY5 protein. It has been proved that the HY5 requires BBX20/21/22 proteins to exert its function in the modulation of transcriptional regulation (Bursch et al., 2020). The existence of the loss-function form of BBX22 detected in this study might provide a route to finetuning the transactivation ability of the HY5 protein.

**Figure 7:**
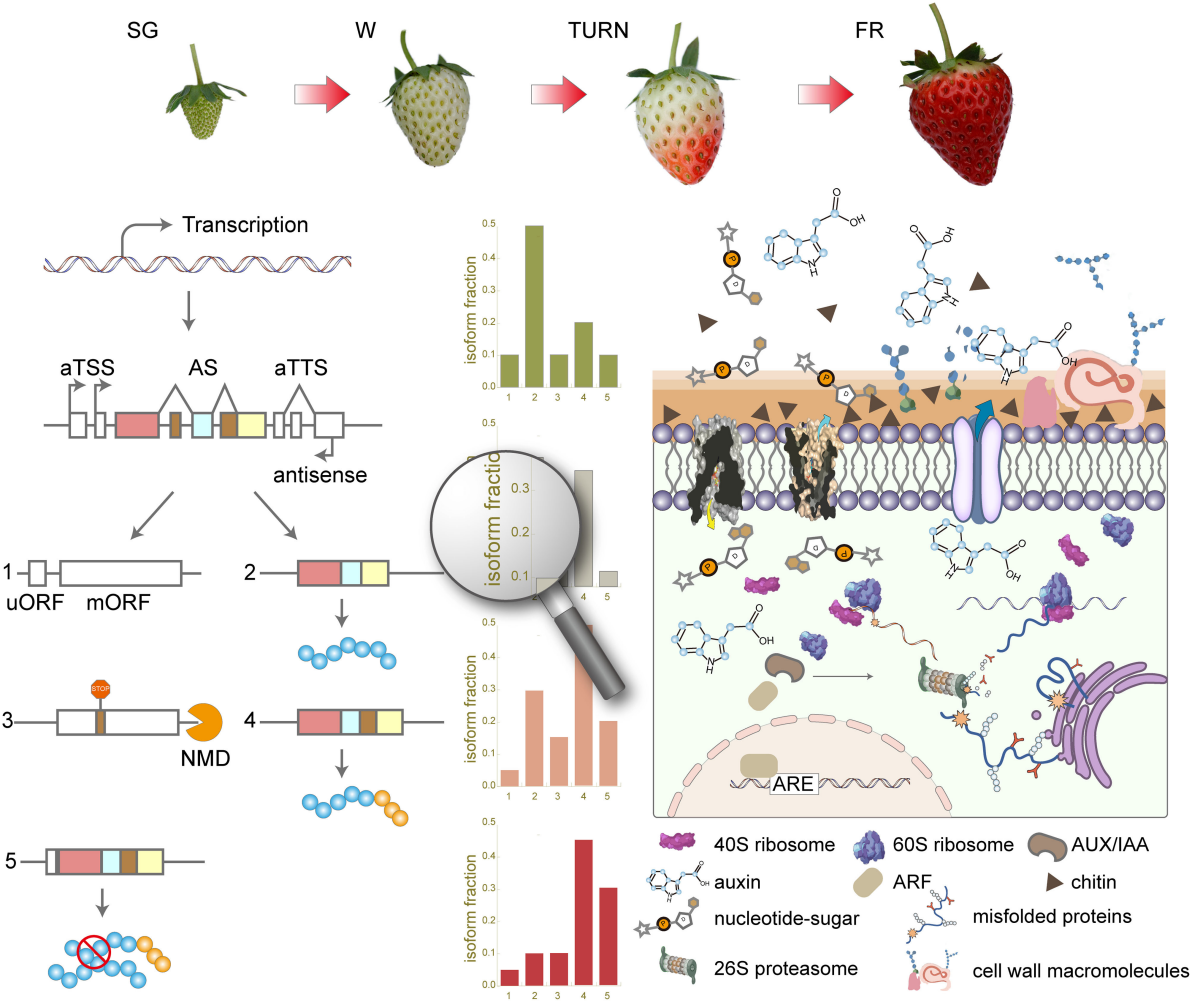
Summary of the transcriptome complexity introduced by alternative transcription start site (aTSS), alternative transcription stop site (aTTS), and alternative splicing (AS) in the strawberry fruit development and maturation. SG, small green; W, white; TURN, turning stage; FR, full red stage; uORF, upstream open read frame; mORF, main open reading frame; NMD, non-sense-mediated mRNA decay; ARF, auxin response factor; ARE, auxin response elements; ERAD, endoplasmic-reticulum-associated protein degradation.

Overall, in this study, we have systematically investigated the transcriptome complexity using long-read ONT techniques along the four successive developmental stages. The full-length cDNA sequencing results unraveled thousands of previously unexplored transcript isoforms. aTSS and aTTS rather than AS accounted for the majority of the complex transcriptome profile in the strawberry fruit. Isoform switches of transcripts from 757 genes were observed, which were associated with protein-coding potential change and domain gain or loss as the main consequences. Those genes with switched isoforms take part in the key processes of maturation at the late maturation stages. Our results provided a new comprehensive overview (Figure 7) of the dynamic transcriptomic landscape during strawberry fruit development and maturation.

## Data Availability Statement

The datasets presented in this study can be found in online repositories. The names of the repository/repositories and accession number(s) can be found below: The ONT full length transcriptome data could be accessible through the CNGB nucleotide sequence archive (https://db.cngb.org/cnsa/home/) with accession number CNP0002170.

## Author Contributions

Conceptualization and funding acquisition: QC and HT. Methodology: XL, WT, QD, and YW. Resources: YLu and XW. Software: QC, YLi, YuZ, and YoZ. Data curation and visualization: QC, ML, XL, and WH. Writing—original draft preparation and project administration: QC. All authors contributed to the article and approved the submitted version.

## Funding

This work was financially supported by the National Natural Science Foundation of China (31972387).

## Conflict of Interest

The authors declare that the research was conducted in the absence of any commercial or financial relationships that could be construed as a potential conflict of interest.

## Publisher's Note

All claims expressed in this article are solely those of the authors and do not necessarily represent those of their affiliated organizations, or those of the publisher, the editors and the reviewers. Any product that may be evaluated in this article, or claim that may be made by its manufacturer, is not guaranteed or endorsed by the publisher.
