# Supplementary material for: Transcriptomic Complexity in Strawberry Fruit Development and Maturation Revealed by Nanopore Sequencing
Source: Front Plant Sci. 2022 Jul 13;13:872054. doi: 10.3389/fpls.2022.872054 (PMC9326444; doi:10.3389/fpls.2022.872054)
Supplement: Supplementary file 1 [file Table_1.DOCX]

**Supplementary table 1** Primers used for validation of AS transcripts and Reverse transcription PCR in this study

| Primer name | Primer sequences ^a^ |
| --- | --- |
| 9g22390.t1_F1 | GGAGACAGGTGCTGACCCTT |
| 22390_231112_R | TACGGAACTTCAAGCTGGTGTATATAAGC |
| T231112_F2 ^b^ | TTGAACAGTGTTTAATGATGGTGCGAT |
| 12g37990.t1_F1 | TCCTCAGATCAATCTCAAGGGTTTCG |
| 37990_861295_R | TGTGCCAGATGGATCAGTGTAGT |
| T861295_F2 | TGTTTCAAGTTGAATATGCCATTGAGGC |
| 13g03460.t1_F1 ^b^ | CTTTCAGATGCACCGTCATTGTAGT |
| 03460_143295_R ^b^ | ACCTTTATCATACTGACACCAGAGCC |
| T143295_F2 | CTTCGCCTTACTGGGTGCCA |
| 20g19920.t1_F1 ^b^ | TCTGTACATCCCTCTGTCTTCTCAGA |
| 19920_t1_t2_R | TGAACAGAGAACGGAACAGCTG |
| 20g19920.t2_F2 | CAACACACGAGACAATATCCAGAACA |
| 22g17110.t1_F1 ^b^ | GAGACTCAACTCTACAAATATGAACTAAGGATATTC |
| 17110_214939_R | ATTAGCCTCAGTAACAGATCCAGTG |
| T214939_F2 | GAGACTCAACTCTACAAGTAATTTCTTCTTTCA |
| qPCR_214939R | GCCTCAATGTGATCAGAACCATATAAC |
| qPCR_17110R | GGAAGAGCTCTCAGGATCATCAG |
| qPCR_actin2F | GCTAATCGTGAGAAGATGAC |
| qPCR_actin2R | AGCACAATACCAGTAGTACG |
| 19920F_exon ^c^ | TGAACAGAGAACGGAACAGCTG |
| 19920R_exon | ATGCCTTCTTCTTCCAATTGCTTCTG |
| 17110F_exon | CAACAACAACAACAAAATTGTGTGTG |
| 17110R_exon | GGAAGAGCTCTCAGGATCATCAG |
| 22390F_exon | GTTCATTCAAGGCATCCAGAAGATGTG |
| 22390R_exon | TACGGAACTTCAAGCTGGTGTATATAAGC |
| 03460F_exon | ATCCAAGCAACTTCGCCTTACTG |
| 03460R_exon | ACCTTTATCATACTGACACCAGAGCC |
| 37990F_exon | GAGCACTCTCTCAATTCACAGTACTC |
| 37990R_exon | TGTGCCAGATGGATCAGTGTAGT |

^a^ The '_vF' primer was used in combination with the one labeled as '_vR'. Primers to amplify the two isoforms of the same gene were marked with same background color. T214939_vF and qPCR_214939R were used for quantitative PCR analysis of isoform TALONT000214939. The primer 22g17110.t1_vF and qPCR_17110R were used as a primer pair to quantify the expression of the isoform FxaC_22g17110.t1.

^b^ These primers spanning the intron which could only anneal to the specific isoform selected. The location of each primer could be found in Figure 3D.

^c^ The primers located on the flanking exons of the tested transcripts corresponding to the products been shown in supplementary figure 4.
