# Supplementary material for: Transcriptomic Complexity in Strawberry Fruit Development and Maturation Revealed by Nanopore Sequencing
Source: Front Plant Sci. 2022 Jul 13;13:872054. doi: 10.3389/fpls.2022.872054 (PMC9326444; doi:10.3389/fpls.2022.872054)
Supplement: Supplementary file 2 [file Table_2.DOCX]

**Supplementary table 2** The primers used to amplify DNA fragment for the plasmid of yeast two hybrid analysis.

| Primer name | Primer sequences (5'-3') ^c^ |
| --- | --- |
| bbx22_F_rec | gagggtgggtcgaatcaaacAATGAAGATACAGTGTAACGTGTGCG |
| bbx22_R_rec | cgaacggtacatcaaaccacTTAGAATTGCCTACGACGTTTTGAGACG |
| HY5_F_rec | tatcgtcgaggtcgaatcaaATGTTGCAAGACCAAGCCAC |
| HY5_R_rec | cgaacggtacatcaaaccacCTAAGATCCCTCGGCATTTGGAC |
| pAD_R | gtttgattcgacccaccctcT |
| pAD_F | gtggtttgatgtaccgttcgTATAGC |
| pDB_R | ttgattcgacctcgacgataCAGTCA |
| pDB_F | gtggtttgatgtaccgttcgTATAATG |
| COP1_F_rec | tatcgtcgaggtcgaatcaaATGGAGGAGTGTTCAACTGGG |
| COP1_R_rec | cgaacggtacatcaaaccacTTAAGCCGCAAGAACCAGGACC |

^c^ The recombination sequences between the donor gene sequence and the vector acceptor sequence were marked as lower-case letters.
