# Supplementary material for: Transcriptomic Complexity in Strawberry Fruit Development and Maturation Revealed by Nanopore Sequencing
Source: Front Plant Sci. 2022 Jul 13;13:872054. doi: 10.3389/fpls.2022.872054 (PMC9326444; doi:10.3389/fpls.2022.872054)
Supplement: Supplementary file 3 [file Table_3.DOCX]

**Supplementary table 3** Evaluation of the transcriptomic Oxford Nanopore Sequencing Raw Data

| Dataset | # reads | # bases | N50 | MaxLength | MeanQscore | Full length ratio (%) |
| --- | --- | --- | --- | --- | --- | --- |
| FRA | 3,982,248 | 3,518,103,552 | 908 | 7,863 | Q11 | 87.88 |
| FRB | 5,077,885 | 4,228,647,683 | 852 | 7,956 | Q11 | 88.68 |
| FRC | 5,175,677 | 4,363,539,202 | 862 | 7,522 | Q11 | 88.29 |
| TA | 5,530,193 | 4,532,170,000 | 839 | 12,295 | Q11 | 89.14 |
| TB | 4,357,046 | 3,534,382,882 | 830 | 8,265 | Q11 | 89.05 |
| TC | 5,284,772 | 4,059,343,004 | 777 | 6,594 | Q11 | 88.94 |
| SGA | 4,632,125 | 3,597,916,528 | 755 | 7,631 | Q11 | 89.33 |
| SGB | 3,546,801 | 2,919,481,445 | 816 | 8,606 | Q11 | 89.32 |
| SGC | 3,143,600 | 2,522,007,285 | 781 | 9,287 | Q10 | 87.35 |
| WA | 3,482,861 | 2,969,605,581 | 868 | 11,846 | Q11 | 89.31 |
| WB | 4,056,389 | 3,290,778,336 | 816 | 7,710 | Q11 | 88.26 |
| WC | 4,395,852 | 3,421,119,169 | 787 | 8,221 | Q11 | 89.26 |
| Total | 52,665,449 | 42,957,094,667 | - | - | - | - |
