# Supplementary material for: Transcriptomic Complexity in Strawberry Fruit Development and Maturation Revealed by Nanopore Sequencing
Source: Front Plant Sci. 2022 Jul 13;13:872054. doi: 10.3389/fpls.2022.872054 (PMC9326444; doi:10.3389/fpls.2022.872054)
Supplement: Supplementary file 9 [file Data_Sheet_1.DOCX]

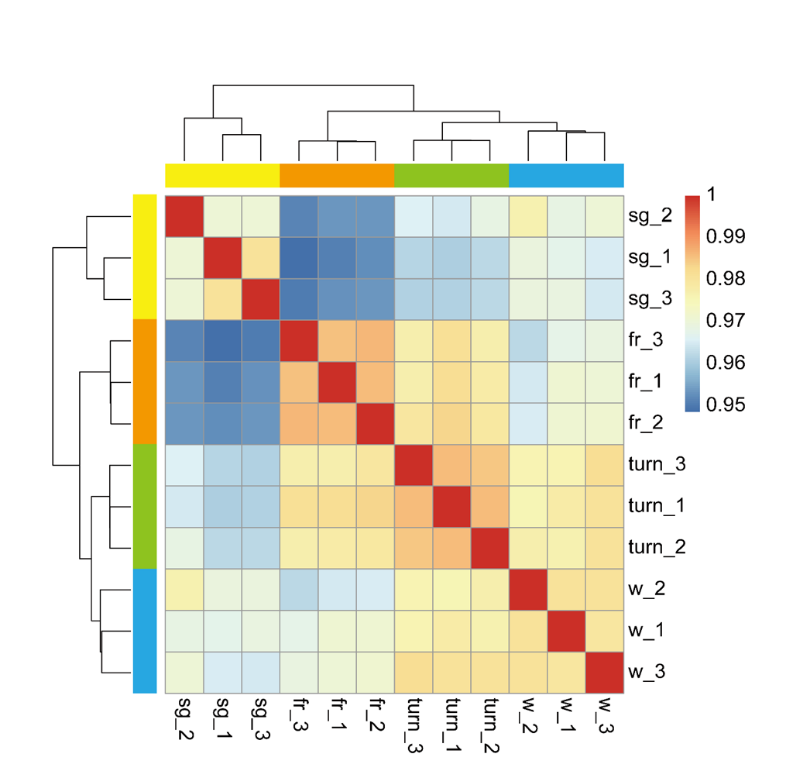


**Supplementary Figure 1.** Heatmap of the Pearson's correlation coefficient of all samples using expression values of all detected genes.


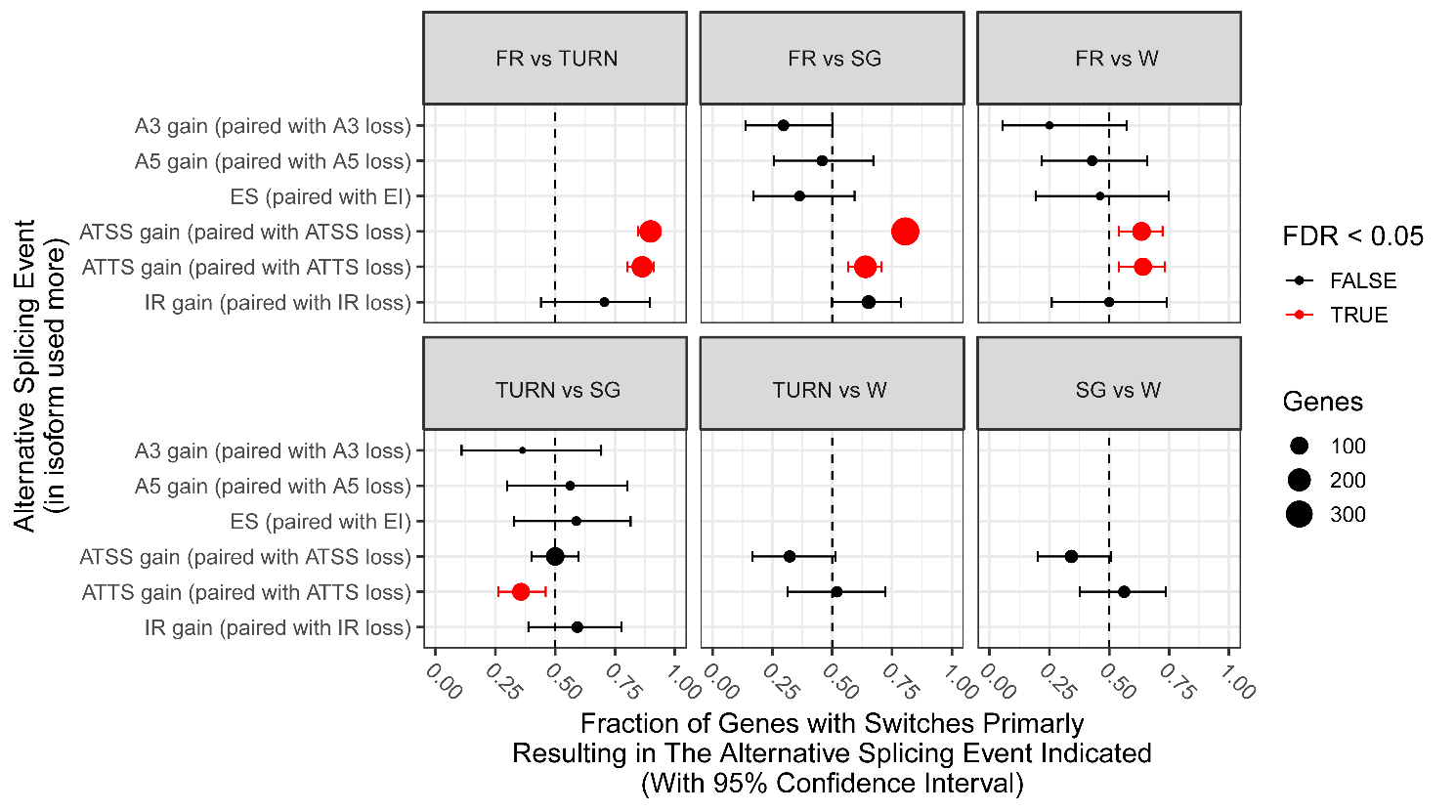


**Supplementary Figure 2.** Alternative splicing shift of the genes with isoform switches in strawberry fruits of different stages.


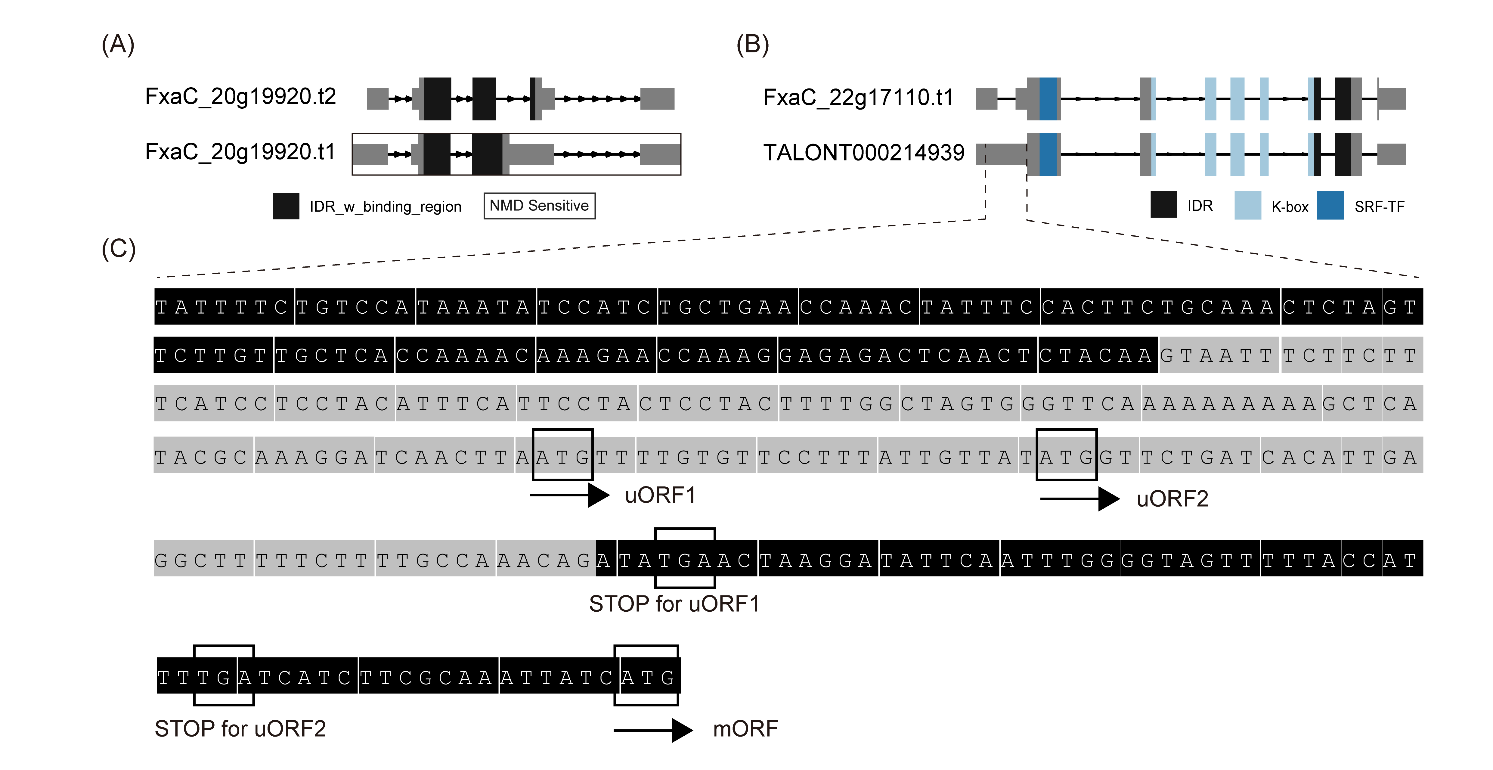


**Supplementary Figure 3.** Functional consequences of AS in two genes with significant isoform switches. (A) Intron retention of FxaC_20g19920 lead to mRNA sensitivity to NMD; (B) Intron retention in the 5' UTR region of FxaC_22g17110; (C) Two uORF features found in the retained intron region of the FxaC_22g17110 transcripts


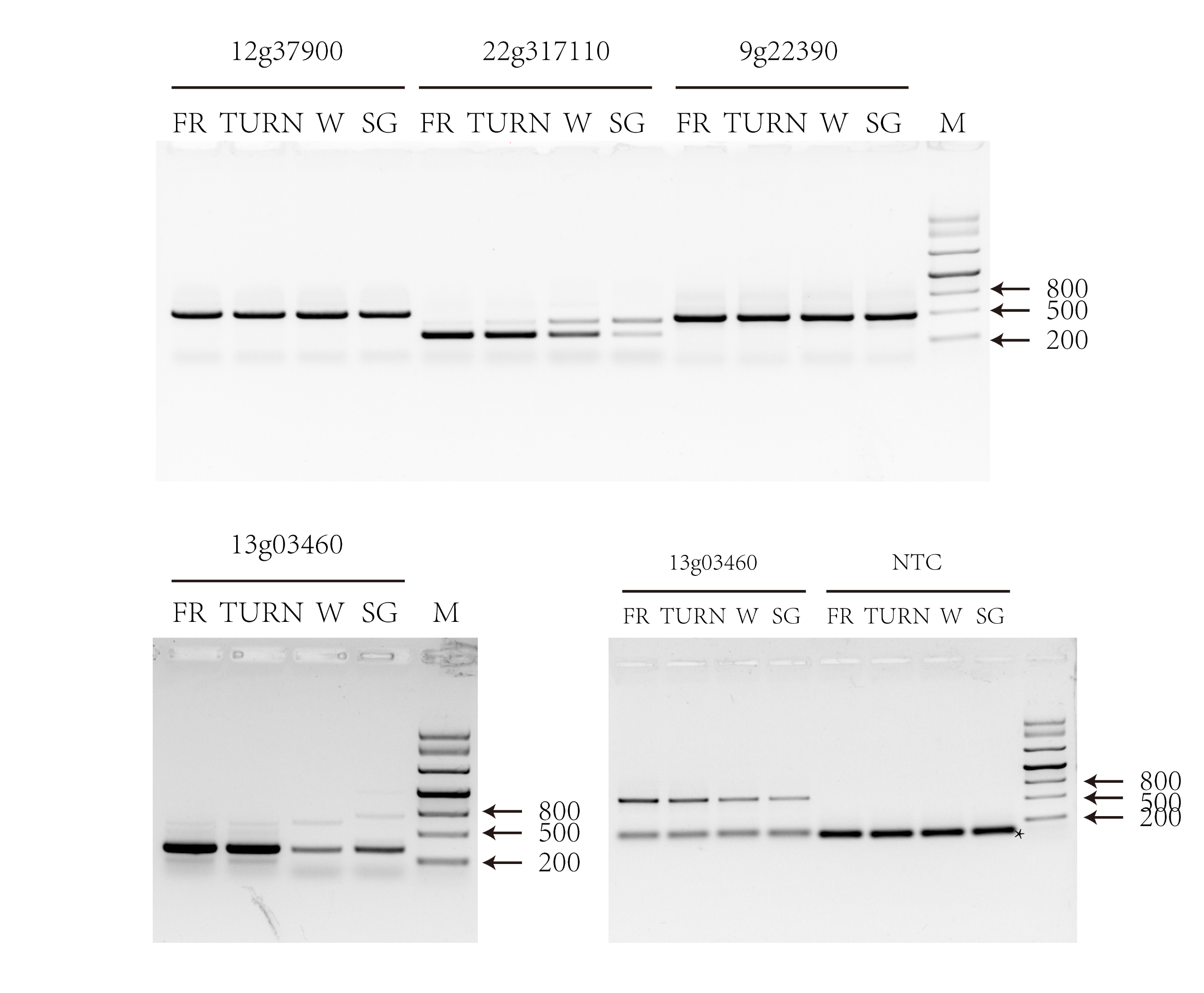


**Supplementary Figure 4**. The RT-PCR amplification products using primers located on exons spanning the retained/skipped exon/exons. The star symbol indicates primer dimer location.
